# DNA Damage Responses and Oxidative Stress in Dyskeratosis Congenita

**DOI:** 10.1371/journal.pone.0076473

**Published:** 2013-10-04

**Authors:** Larisa Pereboeva, Erik Westin, Toral Patel, Ian Flaniken, Lawrence Lamb, Aloysius Klingelhutz, Frederick Goldman

**Affiliations:** 1 Department of Medicine, Division of Hematology Oncology, University of Alabama at Birmingham, Birmingham, Alabama, United States of America; 2 Department of Pediatrics, Division of Hematology Oncology, Children's Hospital of Alabama, Birmingham, Alabama, United States of America; 3 Department of Microbiology, University of Iowa, Iowa City, Iowa, United States of America; Centro di Riferimento Oncologico, IRCCS National Cancer Institute, Italy

## Abstract

Dyskeratosis congenita (DC) is an inherited multisystem disorder of premature aging, cancer predisposition, and bone marrow failure caused by selective exhaustion of highly proliferative cell pools. DC patients also have a poor tolerance to chemo/radiotherapy and bone marrow transplantation. Although critically shortened telomeres and defective telomere maintenance contribute to DC pathology, other mechanisms likely exist. We investigate the link between telomere dysfunction and oxidative and DNA damage response pathways and assess the effects of antioxidants. *In vitro* studies employed T lymphocytes from DC subjects with a hTERC mutation and age-matched controls. Cells were treated with cytotoxic agents, including Paclitaxel, Etoposide, or ionizing radiation. Apoptosis and reactive oxygen species (ROS) were assessed by flow cytometry, and Western blotting was used to measure expression of DNA damage response (DDR) proteins, including total p53, p53S15, and p21^WAF^. N-acetyl-cysteine (NAC), an antioxidant, was used to modulate cell growth and ROS. In stimulated culture, DC lymphocytes displayed a stressed phenotype, characterized by elevated levels of ROS, DDR and apoptotic markers as well as a proliferative defect that was more pronounced after exposure to cytotoxic agents. NAC partially ameliorated the growth disadvantage of DC cells and decreased radiation-induced apoptosis and oxidative stress. These findings suggest that oxidative stress may play a role in the pathogenesis of DC and that pharmacologic intervention to correct this pro-oxidant imbalance may prove useful in the clinical setting, potentially alleviating untoward toxicities associated with current cytotoxic treatments.

## Introduction

The termini of human chromosomes are capped by hexameric DNA repeats called telomeres that protect chromosomes against steady-state attrition and regulate cellular lifespan. Telomeres are bound by a protein complex termed ‘shelterin’ and are maintained by the ribonucleotide enzymatic complex composed of a catalytic component (TERT), an RNA template (TERC), and a number of accessory proteins [1]. Certain mutations residing in telomerase, shelterin and related proteins have been implicated in dyskeratosis congenita (DC) [2]. DC is an inherited premature aging disorder characterized by the triad of skin dyspigmentation, nail dystrophy, leukoplakia, and additionally is associated with bone marrow failure and cancer predisposition [3]. Cells reliant on self-renewal, such as highly replicative tissues and stem cells, require telomere maintenance for long-term survival and are the niches most susceptible in DC (bone marrow, gut, skin). The means by which shortened telomeres elicit cell senescence/death is not completely understood. Under steady-state conditions, telomeres conform to a secondary structure that evades DNA damage surveillance, while shortened and dysfunctional telomeres are thought to engage double-stranded DNA repair mechanisms [4]. These mechanisms include the local deposition of 53BP1/γH2AX initiating a signaling cascade by way of ATM/ATR, CHK1/2 and the eventual activation of the tumor suppressor p53. Continuous telomere attrition in the absence of telomerase will sustain p53 activity leading to replicative senescence or apoptosis.

Dysregulation of p53 may have an underlying role in the pathology of several hematopoietic disorders. In Fanconi's anemia (FA), causative mutations that lie within genes related to DNA repair mechanisms lead to heightened p53 responses that disrupt normal hematopoiesis [5], [6]. Diamond-Blackfan anemia (DBA), characterized by erythropoietic failure, is typically caused by mutations in genes involved in ribosomal biogenesis. The importance of p53 in these diseases can be seen when its expression is experimentally decreased in CD34+ cells, restoring normal *in vitro* and *in vivo* hematopoietic function [6], [7]. The role of p53 activation in DC has also been examined. Gu *et al*. and Kirwin *et al*. evaluated the DNA damage response (DDR) in murine (Dkc1 Δ15) [8] and primary human cells (DKC1, TERT, TERC mutations) [9], and differences were found regarding cellular hypersensitivity to DNA damaging agents. Our lab has previously characterized a heightened DDR in DC fibroblasts, noting the association of short telomeres, subsequent downstream p53 activation, and upregulation of reactive oxygen species (ROS) [10]. ROS could be genetically manipulated by exogenous expression of TERT or knockdown of p53 by shRNA, while the induction of telomere dysfunction in normal cells could increase ROS. Of note, a low oxidative environment partially rescued the proliferative disadvantage in DC cells, suggesting that oxidative stress plays a causative role in suppressing cell proliferation. Together, this data supports a prominent role for the DDR in DC pathology whereupon elevated ROS may have a functional role in carrying out telomere-related cell death.

Herein, we have undertaken studies to further investigate the nature of DDR in primary lymphocytes acquired from members of a DC family (TERC mutation) and whether these cells exhibit increased ‘chemosensitivity’. We provide evidence for a ‘stressed’ phenotype in these cells that may be of direct relevance to DC pathology. Finally, we have for the first time uncovered elevated DDR and ROS in DC lymphocytes that could be rescued, in part, by the antioxidant N-acetyl cysteine (NAC), providing a potential therapeutic avenue for disease manifestations in these patients.

## Materials and Methods

Blood samples were obtained from DC patients or healthy volunteers after written consent in accordance with the principles expressed in the Declaration of Helsinki and the protocols that were approved by the University of Iowa and University of Alabama at Birmingham Internal Review Boards.

### Cells and tissue culture

Cells from DC subjects (n = 5) were obtained with written consent and approval from the University of Iowa Internal Review board. These patients are part of a multigenerational kindred with a deletion of the terminal 74 base pairs of the TERC gene, giving rise to a haploinsufficient, autosomal dominant form of DC [11]. Cells for controls were obtained from healthy volunteers with written consent and approval from the University of Alabama at Birmingham Internal Review board. Mononuclear cell fractions were isolated from whole blood following Histopaque-1077 (Sigma Aldrich) gradient separation and frozen in aliquots. Cells were cultured in complete RPMI-1640 media (10% fetal calf serum, 1000 U/ml penicillin and streptomycin, 20 mM L-glutamine) supplemented with 50 U/ml human interleukin 2 (IL2, Peprotech). Dynabeads Human T-activator CD3/CD28 (Invitrogen Dynal) added at a bead-to-cell ratio of 1∶1 at day 1 was used to stimulate lymphocyte proliferation.

### Assessment of cell proliferation

Cell counts were performed on the ViCell-XR automated cell viability analyzer (Beckman-Coulter). Cell proliferation was expressed as a stimulation index (SI) presenting a fold increase in total cell number relative to the culture starting cell number.

### DNA damaging agents

DNA damage was induced by single exposure to irradiation (XRT) (100–500 cGy) or treatment with Etoposide (10^−5^–10^−7^ M) or Paclitaxel (10^−6^–10^−8^ M) for four days. Cells were irradiated using X-ray irradiation system (X-RAD 320, Precision X-ray Inc. North Branford, CT). Sensitivity to stressor was estimated as ratio of cell number in treated culture relative to untreated culture.

### Apoptosis assay

Basal level of apoptosis was determined after cells were in culture for 5–6 days. XRT-induced level of apoptosis was determined at day 1 after irradiation. Cells were stained for 15 minutes with antibody to AnnexinV-FITC and propidium iodide (PI) using Annexin V-FITC Apoptosis Detection Kit (BD Pharmingen). Flow cytometry was performed with BD FACSCalibur and results were analyzed using CellQuest software.

### Measurement of intracellular ROS

Level of ROS was determined by using Dichlorofluorescin diacetate (DCF-DA, Sigma). Cells collected at indicated times were washed with PBS, and incubated in 1 ml of PBS with 10 uM DCF-DA for 10 minutes at 37°C. After washing twice with PBS, cells were subjected to FACS analysis. ROS levels were quantified by recording the mean fluorescent intensity (MFI).

### Western blotting

Standard Western blotting techniques were used as previously described [10], [12]. Briefly, cells were pelleted and lysed with Complete Lysis-M buffer (Roche). Whole cell extracts were subjected to SDS-PAGE electrophoresis, transferred to a nitrocellulose membrane, and stained with antibodies to: p53 (Calbiochem), p53S15 (serine15 phosphorylated p53, Cell Signaling), p21^WAF^ (BD Pharmingen), and actin (Santa Cruz), then a secondary antibody conjugated with HRP (Santa Cruz).

### Antioxidant treatment

NAC (Sigma Aldrich) was added directly to cell cultures at varying time points and used at a final concentration of 10 mM.

### Statistical analyses

Student's t-test was applied to assess statistical significance between two groups of data, and calculated p-values are reported. Analysis was performed using Graph Pad Prizm software. Error bars within graphs are representative of the standard deviation of DC or control samples in each experiment.

## Results

### DC lymphocytes have impaired *in vitro* cell growth and increased sensitivity to DNA damaging agents

**Figure 1:**
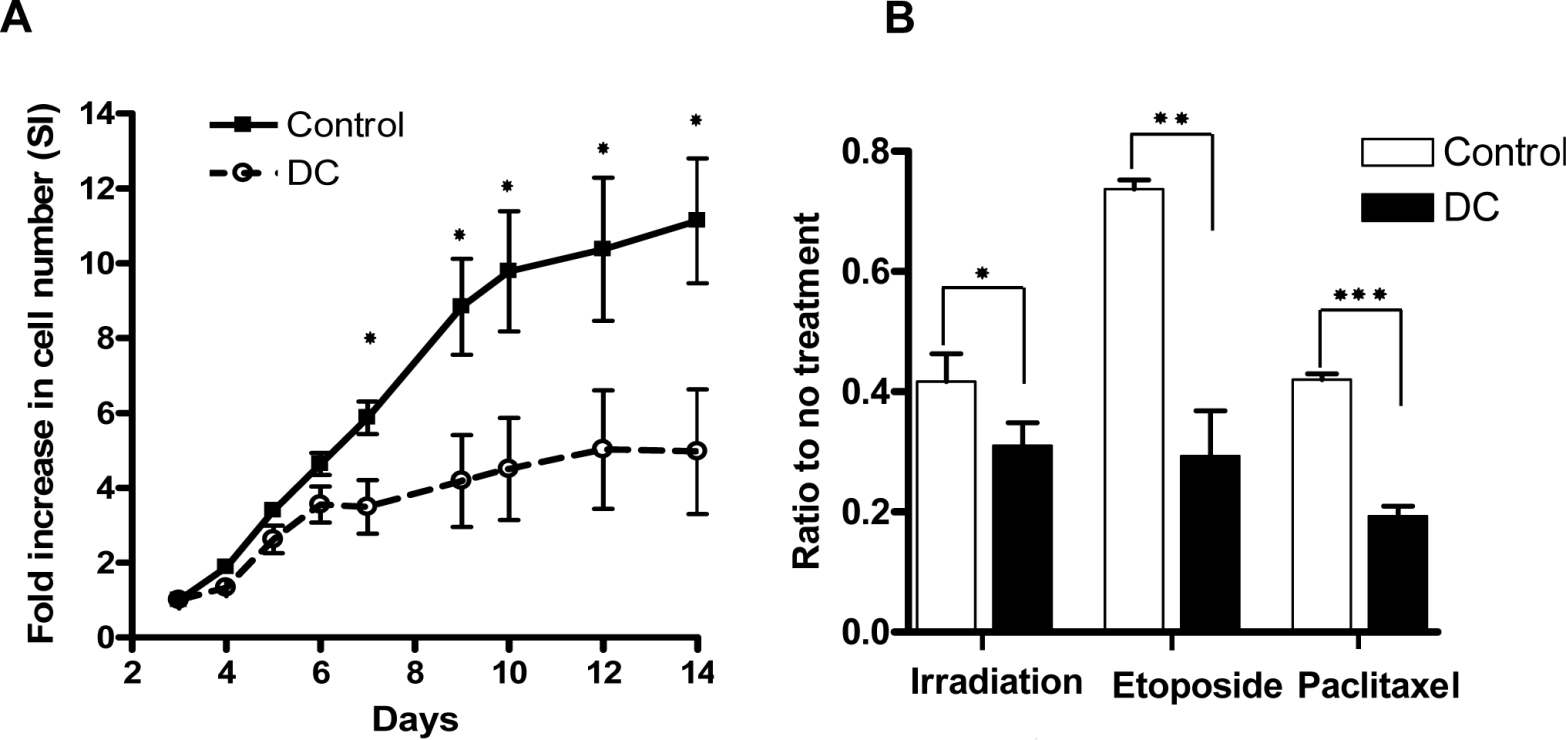
Impaired growth of DC lymphocytes in cell culture. (A) Control (n = 5) and DC (n = 5) lymphocytes were stimulated with CD3/CD28 beads at day 1 and cultured in IL-2 supplemented medium. The stimulation index (SI) is calculated as a fold increase in cell number relative to the starting cell number. Statistically significant difference in proliferation of DC versus control lymphocytes was noted starting from day 7 (*p<0.01). (B) Increased growth sensitivity of DC lymphocytes to irradiation (XRT) and chemotherapy. Control (n = 4) and DC (n = 5) cells were treated with XRT (5 Gy) and proliferation was assessed two days later. Alternatively cells were treated with Etoposide (10^−5^ M) or Paclitaxel (10^−6^ M) for four days and assessment of cell growth was done two days after treatment. Data is presented as a ratio of cell numbers in treated versus their respective untreated culture controls. A statistically significant decrease in DC cell growth compared to controls was determined after XRT (*p<0.05), or after treatment with Etoposide (**p<0.01) and Paclitaxel (***p<0.0005).

It has been previously reported that primary skin fibroblasts and keratinocytes isolated from DC patients have impaired growth and function [10], [13], [14], [15], [16]. In addition, lymphocytes from DC patients have a senescent phenotype with a reduced proliferative capacity and altered mitotic profile [17] while CD34+ hematopoietic progenitor cells have a greatly reduced colony forming ability [18]. These results are consistent with the clinical phenotype of DC that includes marked mucocutaneous abnormalities, nail dystrophy, immune dysfunction, and bone marrow failure. Several years ago our group established a frozen tissue repository of TERC deficient DC cells, and marked telomere shortening was noted in lymphocytes from all DC subjects (less 1% of age matched controls) [18]. Here, initial experiments were carried out to validate the proliferative defect of cultures established from frozen/thawed peripheral blood mononuclear cells (PBMC) of DC subjects compared to similarly treated age-matched healthy control cells. The initial expansion rate of DC cells using T-cell activating conditions (CD3/CD28 beads) was similar to control samples after five days in culture, increasing 2–4 fold (Fig. 1A). Of note, immunophenotyping at day 5 consistently showed that greater than 95% of cells in stimulated culture were CD3 positive (data not shown). While control cells continued robust expansion for two weeks (SI range 8–12 at day 14), DC cell growth plateaued at day 9 (SI range 3–5), and remained constant until day 14. These findings confirm a proliferative disadvantage in stimulated DC lymphocytes.

To determine if the intolerance of chemotherapy in DC patients is related to an intrinsic DNA repair defect, lymphocytes from five DC subjects and age-matched controls were treated with Paclitaxel (anti-mitotic agent and microtubule inhibitor), Etoposide (topoisomerase II inhibitor and DNA damaging agent), or ionizing radiation (induction of double-strand DNA breaks). After 3–5 days following exposure to radiation (XRT), DC lymphocytes had a statistically significant diminished proliferation relative to control cells (p<0.05). Similarly, DC lymphocytes exposed to Paclitaxel or Etoposide displayed an even greater sensitivity, with statistically significant decreases in stimulation indices (p<0.01 and p<0.0005) (Fig. 1B). This data suggests that DC cells are particularly sensitive to DNA damaging agents, consistent with clinical observations.

### Increased apoptosis, ROS and p53 expression in DC lymphocytes

**Figure 2:**
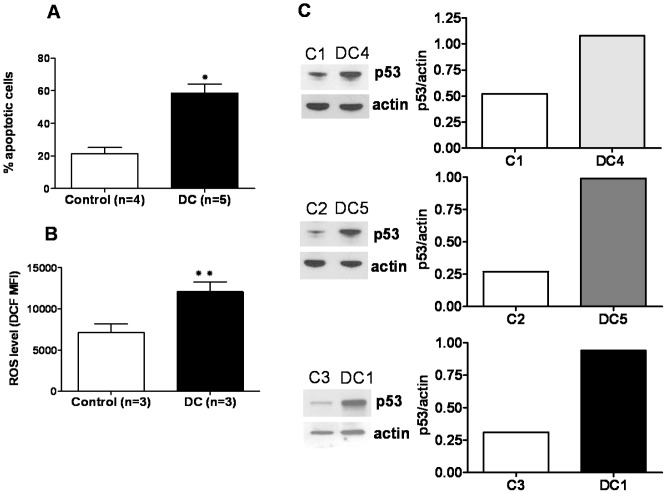
Elevated levels of apoptosis, reactive oxygen species (ROS) and p53 in DC lymphocytes. Control and DC lymphocytes were cultured with CD3/CD28 beads in IL-2 supplemented media for 5–6 days. (A) The percentage of apoptotic cells, as determined by flow cytometry after co-staining cells with annexin-V antibody and propidium iodide, was increased in DC cultures (*p<0.001). (B) ROS level, as determined by flow cytometry after incubation of cells with DCF and presented as the mean fluorescent intensity (MFI), was increased in DC cells (** p<0.03). (C) p53 expression, as determined by Western blot and quantified using Image J densitometry software, was increased in DC cells (p<0.05). Representative data is shown for three different DC subjects and controls.

Previous studies indicate primary DC lymphocytes have increased apoptosis in short and long-term cultures [17] [9]. Experiments were thus undertaken to determine if there was an association between decreased proliferative capacity in DC cells and stress related markers, including apoptosis, ROS, and p53 expression. In DC cultures from five different subjects, the percentage of apoptotic cells increased over a two week time course, and at each time point repeatedly demonstrated 2–3 fold more apoptotic cells compared to controls. As noted in Figure 2A, a statistically significant increase in apoptotic cells was seen in stimulated DC cultures compared to controls after 5 days (p<0.001). Elevated levels of ROS have also been reported in DC fibroblasts [10]. Similar to apoptosis data, steady state ROS levels in cell culture under log phase growth were nearly two-fold higher in DC cells relative to controls (p<0.03, Fig.2B). Finally, studies were carried out to determine whether increased apoptosis and ROS levels were also accompanied by changes in p53 expression. Under the same culture conditions, p53 levels were, in general, up-regulated 2–4 fold in DC cells relative to control samples (p< 0.05, Fig. 2C). In summary, DC lymphocytes demonstrated a “stress” phenotype characterized by elevated apoptosis, ROS and p53 expression.

### Radiation-induced levels of apoptosis, ROS and DDR marker expression in DC lymphocytes

**Figure 3:**
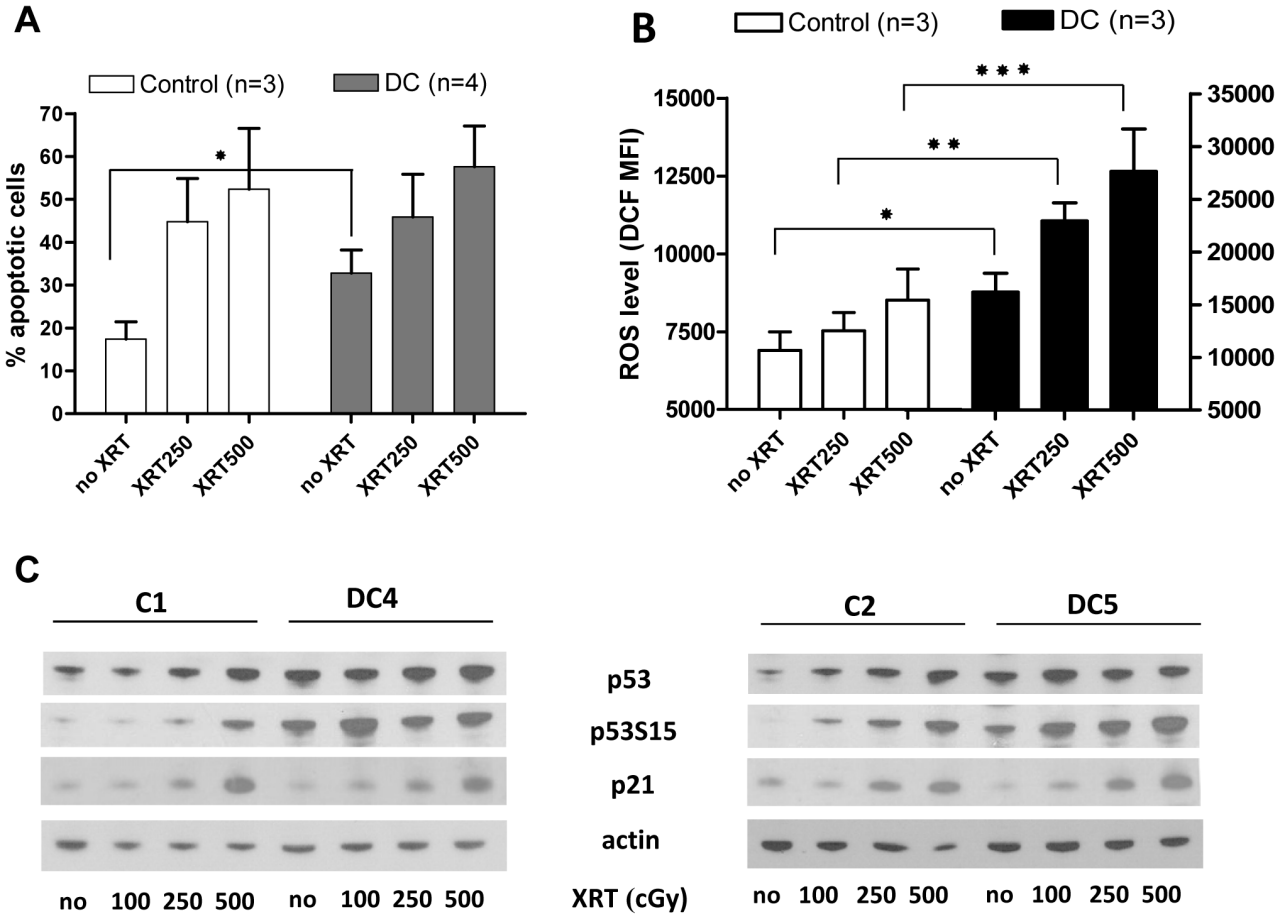
Irradiation-induced levels of apoptosis, ROS and DDR markers in DC lymphocytes. Control and DC cells were subjected to increasing doses of irradiation (0–500 cGy)and 24 hours later assayed for the percentage of apoptotic cells (A) and level of ROS (B). Statistically significant differences were noted between DC and matched controls (*p<0.02, **p<0.003, ***p<0.01) and non-irradiated and irradiated DC (p<0.05). (C) DDR protein expression, including p53, p53S15 and p21 were assessed by Western blotting, and representative blots of five separate experiments are shown.

To further define the relationship between “proliferative stress” in DC cells and the observed cellular sensitivity to DNA damaging agents, DC and control lymphocytes were exposed to non-lethal doses of ionizing radiation (250 and 500 cGy). 24 hours post-treatment, cells were assessed for apoptosis, ROS production and DDR signaling. Consistent with our earlier finding (Fig 2A), non-irradiated DC cells demonstrated a statistically significant increase (p<0.02) in apoptosis relative to non-irradiated controls. However, only a minimal difference in apoptosis was noted in irradiated DC cells relative to irradiated controls (Fig. 3A). Similarly, steady state (non-irradiated) levels of p53 and phosphorylated p53S15 were upregulated in DC lymphocytes relative to controls. However, in non-irradiated cells, p21 expression was not upregulated and was similar to control cells (Fig. 3C). With irradiation, the magnitude of expression of p53 and p53S15 in DC cells did not markedly increase, though a dose dependent response was noted in control cells. In contrast, p21 protein expression was upregulated following irradiation in both DC and control cells, suggesting a p53-independent mechanism of p21 regulation. While radiation had a minimal effect on increasing ROS in control cells, we found irradiated DC cells had a statistically significant (p<0.02) increase in ROS production relative to irradiated control cells (Fig. 3B). In addition, we also found an increase in ROS production that was radiation-dose dependent in DC cells (p<0.05) (Fig 3B). Together, these data suggest the magnitude of p53 expression and ROS levels may influence DC cell survival in response to various stressors and DNA damaging agents otherwise tolerated by normal cells.

### N-acetyl cysteine (NAC) rescues growth disadvantage and decreases apoptosis, ROS, and DDR markers in DC lymphocytes

**Figure 4:**
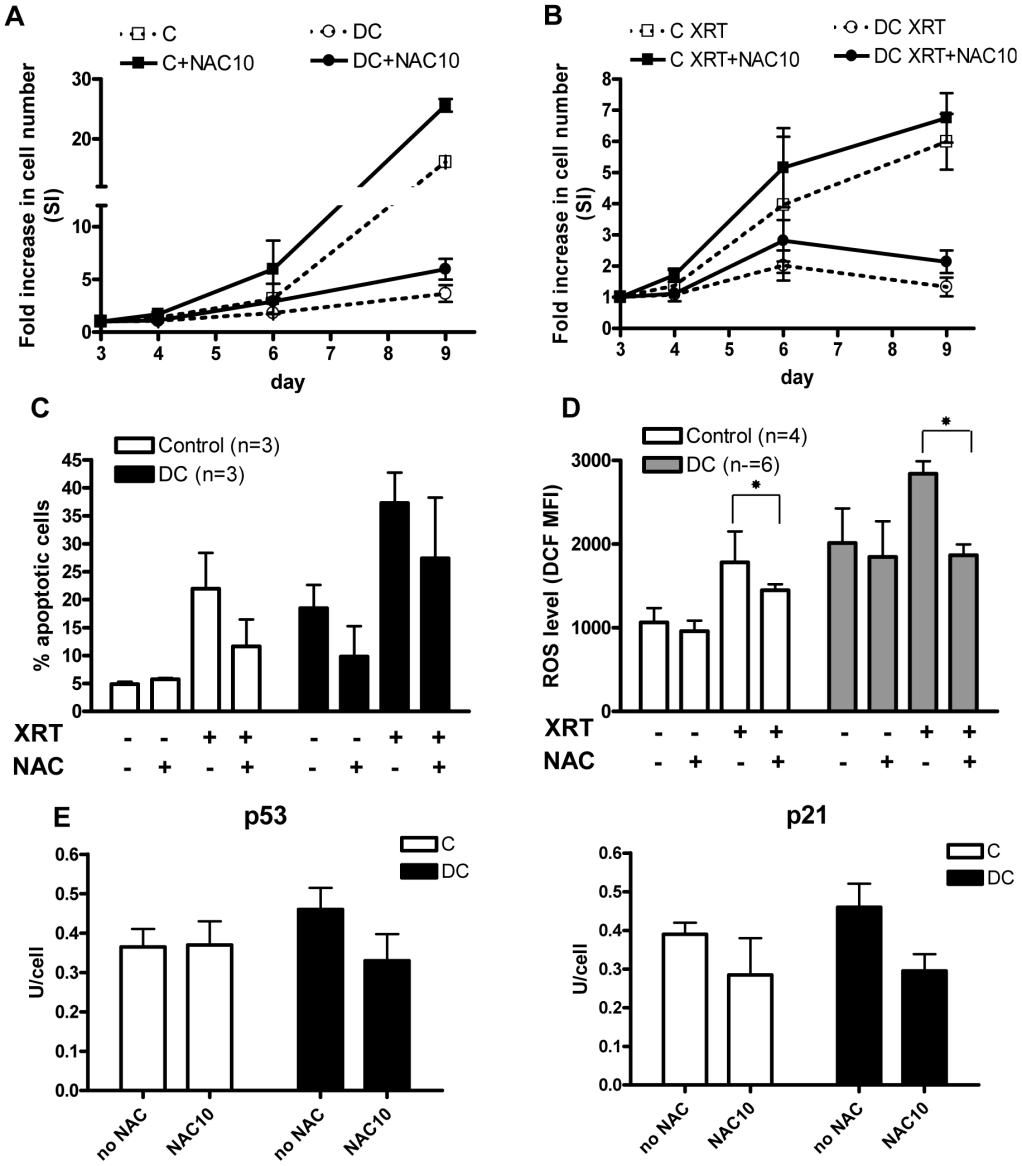
NAC treatment alleviates growth inhibition, apoptosis, ROS and DDR mobilization in DC lymphocytes. DC (n = 5) and control cells (n = 5) were cultured in the presence or absence of 10 mM NAC in growth media and irradiated with 500 cGy at day 5. Growth curves of non-irradiated (A) and irradiated (B) cultures are plotted. The percentages of apoptotic cells (C) and levels of ROS (D) were determined one day pre and post XRT, and statistical significance is noted (*p<0.05). (E) Expression of p53 and p21 was determined by Western blot from total cell lysates at 50,000 cells collected 3 hours after NAC addition. Data presented in arbitrary units per cell and averaged for control (n = 2) and DC (n = 4) samples.

Given the increased steady-state and radiation-induced levels of ROS, *in vitro* experiments were undertaken to determine whether antioxidant therapy could ameliorate impaired growth, ROS generation, and DDR signaling in DC lymphocytes. NAC is a pharmacological antioxidant used in a number of clinical conditions and is FDA approved as an antidote for acute acetaminophen toxicity. Preliminary dose response experiments were performed in control cells and a concentration of 10 mM was chosen, as this dose was deemed to be non-toxic and was consistent with pharmacologic dosing. As shown in Figures 4A and 4B, NAC resulted in an improvement in growth of control and DC lymphocytes in both non-irradiated and irradiated cultures. An increase in cell number in DC and control cultures treated with NAC was observed during all time points tested, though this was not statistically significant (p>0.05). Next, we examined the effect of NAC on levels of apoptosis and ROS in cultured lymphocytes. Without NAC, radiation increased the percentage of apoptotic cells in control and DC samples (Fig. 4C). In non-irradiated cells, NAC appeared to have a greater effect on apoptosis in DC relative to control cells. Importantly, NAC reduced apoptosis 1.3–2 fold in irradiated control and DC cultures. NAC did not substantially decrease ROS in non-irradiated control and DC cells, though it did have a statistically significant effect in irradiated control and DC cells (p<0.05), as noted in Fig 4D. Lastly, we assessed whether NAC modulated expression of p53 and p21. As indicated in Fig 4E, NAC did not have an appreciable effect on p53 expression in control cells, though a slight decrease was noted in DC cells. Similarly, NAC decreased the expression of p21 in control and DC cells. However, neither of these experiments reached statistical significance. These data suggest that NAC may have a protective effect on cells by decreasing oxidative stress and that this effect may be most appreciable in cells with a stress phenotype, as noted in DC cells.

## Discussion

DC is a disorder of telomere dysfunction and often manifests in tissues with high proliferative capacity, including skin, gastrointestinal tract, immune system, and bone marrow. Coincident with the clinical triad of leukoplakia, skin dyspigmentation, and nail dystrophy, *in vitro* studies of human DC skin cells (fibroblasts and keratinocytes) have consistently demonstrated a reduced proliferative potential [13] [15]. Bone marrow failure is frequently noted in DC, though variable in onset, reflecting the essential role of telomerase and telomere length maintenance in hematopoietic stem cells and progenitors. The development of marrow failure can be partially explained by a lower frequency of long-term colony-initiating cells in DC marrow [18]. However, there is evidence from telomerase knockout mice that a defective stem cell niche may play a role [19]. Immune abnormalities have also been described in DC [17] due in part to the requirement in innate immunity of lymphocytes ability to undergo extensive expansion. To better understand this process, we carried out our experiments using lymphocytes that were obtained from DC subjects with TERC deficiency. Over a two week time course in culture conditions of CD3/CD28 activation, a growth deficiency was noted relative to controls, indicating an underlying proliferative defect. Though stimulation conditions were different, similar findings were noted by Kirwan et al, and growth inhibition was not influenced by DC mutation status [9]. Of note, we also found a significant decrease in proliferation in DC cells, relative to controls, after exposure to Etoposide, Paclitaxel, and XRT, suggesting an increased sensitivity to DNA damaging agents.

The association of bone marrow failure and malignancy with DC has resulted in many patients undergoing chemotherapy treatments and hematopoietic stem cell transplantation (HSCT) [3]. DC patients have also been noted to have an increase in transplant-related morbidity using standard myeloablative preparative regimens, leading to the successful development of reduced intensity regimens [20] [21]. This is consistent with our *in vitro* finding where lymphocytes have an increased sensitivity to cytotoxic agents and is somewhat suggestive of a DNA repair defect, similar to that noted in FA. The “hyper-sensitivity” of FA patients to cytotoxic agents is well documented, and similar to DC, less intense BMT preps are now the standard for FA patients with aplastic anemia [22], [23]. Of note, while abnormal sensitivity of lymphocytes to the clastogens diepoxybutane (DEB) and mitomycin is a diagnostic test for FA, TERC deficient DC lymphocytes subjected to these agents did not show an increase in chromosomal breakage rates (data not shown).

Evidence supporting the relationship between telomere dysfunction, DDR, and p53 activation continues to accumulate [4] [24] [25]. This relationship has been verified in DC cells by our group [10] and others and in a mouse model of DC [8], [26]. By engaging DDR, shortened telomeres activate p53, which is a key determinant in cell fate decisions. Attenuating p53 through different mechanisms rescues some of the defects associated with short telomeres, further supporting the role of p53 in telomere-related pathologies [27]. The role of p53 in hematopoiesis is complex, on the one hand being necessary for inhibition of malignancy but on the other being potentially antagonistic to normal proliferation. Although required for maintaining long-term proliferative capabilities via quiescence, chronic p53 activity taxes the hematopoietic stem cell pool leading to cell depletion [28]. Recently, heightened HSC p53 activity was found in the human bone marrow failure syndromes FA [6] and DBA [7], leading to premature senescence and depletion of progenitor cells. In addition, p53 was found to be important for mediating the effects of defective RNA processing in the hematopoietic cells of a dyskeratosis congenita zebrafish model [29]. While unable to directly assess p53 levels in DC marrow, we did note an increase in steady-state DDR markers p53 and p53S15 in DC lymphocytes (Fig 2). This contrasts with that reported by Kirwan et al [9], where expression of DDR proteins γH2AX and 53BP1 were found to be no different than controls. Reasons for these differences may be partially explained by the specific genotypes of the cells that were used but also by the use of different markers to assess DDR. All primary cells in this study were obtained from patients with an autosomal dominant form of DC where a TERC deletion results in haploinsufficiency. Further studies are needed to determine whether our findings represent a general feature of DC cells with variable genetic backgrounds. Consistent with our data, Kirwan et al. did note increases in telomere dysfunction-induced foci in DC cells, which is indicative of an enhanced DDR. Interestingly, Gu et al. demonstrated a consistent increase in γH2AX, p21, and p53 expression after Etoposide treatment in dyskerin mutant mouse embryo fibroblasts compared to wild-type cells [8].

Despite notable differences between TERC DC and control lymphocytes with respect to steady-state levels of DDR proteins, we were unable to demonstrate further increases in p53 expression post-irradiation (Fig 3C). We did, however, note a dose-dependent increase in apoptosis and ROS in DC cells, suggesting that DC cells exist at a heightened level of stress. DC cells tolerate this DNA damage less than controls and more readily enter into apoptosis, likely through p53-dependent and p53-independent mechanisms.

In addition to an increased expression of p53 and apoptosis, levels of ROS were also significantly increased in DC lymphocytes (relative to controls) after several days in stimulated culture and following exposure to radiation (Fig 2B and 3B). ROS generation is a natural byproduct of many cellular reactions and required for some physiologic responses such as T-cell activation [30], yet in excess it can be detrimental [31]. The implications of elevated ROS in DC lymphocytes may have bearing on DC-related pathogenesis. Previous work has suggested the hematopoietic compartment is maintained in a low oxidative environment and increases in ROS within this niche severely compromises the long-term replicative capacity of these cells [32]. Elevated ROS is not specific to DC and has been described in FA [33]. Of note, certain pathologic findings in FA (skin dyspigmentation, endocrine abnormalities and malformations) have been attributed to a pro-oxidant state [34], [35]. Our data is consistent with Richter *et al*, where a direct correlation was noted between telomere shortening, ROS levels, and p53 activity [35].

Given our current and previous findings that ROS may be involved in DC pathogenesis, we examined the potential ameliorative effects of the antioxidant agent NAC on DC lymphocytes. We focused on NAC due to its low toxicity profile, as well as recent data indicating that NAC effectively rescued a growth disadvantage in fibroblasts and stem cells within a murine model of Dkc1 (Δ15) [36]. NAC can also partially correct stem cell defects in other premature aging models such ATM-deficiency or in mice lacking transcription factors FoxOs 1, 3, and 5 [37] [38]. NAC has been used successfully, either singly or in combination with other agents, to treat idiopathic pulmonary fibrosis. It is unclear whether NAC is mediating its effect through anti-oxidant or mucolytic properties [39] [40].

At a pharmacologic concentration of 10 mM, we found that NAC was able to decrease basal and radiation-induced levels of ROS, as well as partially rescue cell growth and decrease apoptosis. Thus, reduction of ROS through NAC or other agents might provide a means to treat several different forms of bone marrow failure. Interestingly, we found that short exposure (3 hours) of DC lymphocytes to NAC also resulted in a decrease in p53/p21 expression. While this result might suggest that increased ROS lies upstream of p53 activation, it is also possible that the presence of ROS caused by dysfunctional telomeres may create a positive feedback loop where increased ROS leads to further erosion of telomeres thereby increasing p53 and ROS. A similar model has been proposed for the establishment and maintenance of cellular senescence[41]. The mechanism to account for how p53 activation might cause an increase in ROS is not entirely clear but evidence points to a pathway that involves some aspect of mitochondrial dysfunction through p53-mediated modulation of mitochondrial biogenesis genes or serial signaling involving the TGFβ pathway [27], [41].

In summary, the current study was set up to more fully characterize the molecular basis for proliferative defects in DC lymphocytes. We demonstrated the proliferative defect in DC cells was further pronounced in the setting of cytotoxic agents, suggesting DC patients may be more prone to systemic toxicities associated with these agents. In addition, DC lymphocytes expressed a “stress phenotype” as noted by increased apoptosis, DDR protein expression, and ROS under *in vitro* stimulated culture conditions. This “phenotype”, as well as the defect in proliferation, was partially corrected by the antioxidant NAC. While there is no published data on the efficacy of antioxidants in DC patients, we have noted cutaneous improvements in several DC patients treated with oral and topical Vitamin E (unpublished observations). Experiments designed to pharmacologically suppress ROS in cells harboring telomere dysfunction may provide important insight towards diminishing the toxicity associated with myeloablative treatment in DC patients as well as age-related pathologies in the general population.
